# Evolving trends and burden of idiopathic epilepsy among children (0–14 years), 1990–2021: a systematic analysis for the Global Burden of Disease study 2021

**DOI:** 10.3389/fneur.2025.1548477

**Published:** 2025-03-17

**Authors:** Fulai Tu, Zhengcheng Tu, Xinrui Jiang, Meng Zhao, Wei Li, Chunfeng Wu, Pingmin Wei

**Affiliations:** ^1^Department of Epidemiology and Health Statistics, School of Public Health, Southeast University, Nanjing, Jiangsu, China; ^2^Olin Business School, Washington University in St. Louis, MO, United States; ^3^Department of Neurology, Children’s Hospital of Nanjing Medical University, Nanjing, China; ^4^Department of Clinical Research Center, Children’s Hospital of Nanjing Medical University, Nanjing, China

**Keywords:** idiopathic epilepsy, children, Global Burden of Disease, trends, systematic analysis

## Abstract

**Objective:**

This systematic analysis aims to elucidate the trends and burden of idiopathic epilepsy among children aged 0 to 14 from 1990 to 2021, utilizing Global Burden of Disease (GBD) 2021 data to explore demographic and geographical variations, highlight progress, and identify ongoing challenges.

**Methods:**

Data were sourced from the GBD 2021 database, focusing on children aged 0–14. Annual absolute numbers and age-standardized rates for incidence (ASIR), prevalence (ASPR), mortality (ASMR), and disability-adjusted life years (ASDR) of idiopathic epilepsy were retrieved. Joinpoint regression analyses assessed changes over time, calculating average annual percentage change (AAPC) statistics. Data collation and visualizations were conducted using R software, with statistical significance established at a *p*-value threshold of 0.05.

**Results:**

In 2021, there were 1,227,191 incident cases, 6,095,769 prevalent cases, 3,564,497 DALYs, and 18,171 deaths due to idiopathic epilepsy globally. The ASIR increased by 0.27% from 55.74 to 61.35 per 100,000 population from 1990 to 2021. In contrast, ASPR (AAPC = −0.03), ASMR (AAPC = −1.60), and ASDR (AAPC = −1.01) all decreased. Regionally, the low-middle SDI region had the highest burden, while the high SDI region had the highest ASIR and ASPR. The low SDI region experienced the highest ASMR and ASDR. Significant regional variations were noted, with the African Region exhibiting the highest ASIR and ASDR, while the Western Pacific Region had the lowest. Nationally, substantial variations were observed across 204 countries, with notable differences in ASIR, ASPR, ASMR, and ASDR.

**Conclusion:**

Despite overall declines in ASPR, ASMR, and ASDR, the slight increase in ASIR and regional disparities highlight ongoing challenges. Low and low-middle SDI regions continue to bear a higher burden, underscoring the need for targeted interventions and improved healthcare access. Future efforts should focus on strengthening healthcare systems, enhancing diagnostic and treatment capabilities, and increasing awareness, particularly in resource-limited regions.

## Introduction

Epilepsy is one of the most common neurological disorders affecting children worldwide (1), and is characterized by recurrent seizures that can lead to various neurobiological, cognitive, psychological, and social challenges (2). Idiopathic epilepsy, characterized by an absence of identifiable structural or metabolic cause, comprising a significant proportion of these cases (3, 4). Epilepsy is a chronic brain disorder marked by a lasting tendency to produce unprovoked seizures, the global burden of idiopathic epilepsy among children has substantial implications for healthcare systems, families, and societies, often necessitating long-term medical care, social support, and educational accommodations (5). Over the past three decades, from 1990 to 2021, advancements in diagnostic techniques, treatment modalities, and public health interventions have influenced the epidemiology of idiopathic epilepsy in children (6). Despite these advancements, disparities in healthcare access and treatment outcomes persist across different regions, emphasizing the need for comprehensive and updated data to inform policy and resource allocation.

The GBD Study provides a robust framework for analyzing the trends and burden of various health conditions (7–9), including idiopathic epilepsy (10–12). This systematic analysis utilizes the GBD dataset from 1990 to 2021 to comprehensively understand the epidemiological landscape of idiopathic epilepsy in children. The study analyzes prevalence, incidence, and disease burden using disability-adjusted life years (DALYs) to capture demographic and geographic shifts, track progress, and identify ongoing challenges in managing epilepsy globally. Analyzing these trends helps identify regions where interventions are most urgently needed, enabling the optimization of resource allocation (13). Additionally, understanding the impact of preventive strategies and advancements in treatment can guide targeted actions to reduce disease burden, particularly in regions with substantial unmet needs. Highlighting both progress and existing gaps in addressing idiopathic epilepsy in children emphasizes the importance of evidence-based strategies to bridge regional disparities, ultimately striving for more equitable and effective health outcomes worldwide. This comprehensive approach not only advances the field of epilepsy research but also strengthens public health frameworks, paving the way for future studies and policies that more effectively address this condition.

## Materials and methods

### Study data

Data on Idiopathic epilepsy were obtained from the GBD study 2021 database^1^ (14). This database provides the most comprehensive assessment of disease burden across 204 countries and territories form 1990 to 2021. We extracted data for age-standardized incidence rates (ASIR), age-standardized prevalence rates (ASPR), age-standardized mortality rates (ASMR), and disability-adjusted life-years rates (ASDR). The GBD estimates are based on systematic reviews, population based surveys, and statistical modeling, ensuring robust and comparable results. Ethics approval and informed consent were not required, as the data are publicly available and de-identified.

### Categorization of locations by Socio-demographic Index (SDI) and health regions

To facilitate comparative analyses, we classified the 204 countries into five Socio-demographic Index (SDI) quintiles The SDI serves as a summary measure that places countries or geographical areas along a development spectrum, categorizing them as low, low-middle, middle, high-middle, and high. In addition to SDI categorization, countries were grouped into six major health regions for comprehensive analysis: African Region, Eastern Mediterranean Region, European Region, Region of the Americas South-East Asia Region and Western Pacific Region. This dual categorization enhances the understanding of global health disparities and supports the development of targeted interventions tailored to the specific needs and challenges faced by each country and region. By employing these classifications, we can better address health issues and allocate resources effectively to improve health outcomes worldwide.

### Statistical analysis

Temporal trends in incidence and mortality from 1990 to 2021 were analyzed using Joinpoint Regression Analyses, which identifies significant changes in trend over time and computes average annual percentage change (AAPC) statistics and corresponding 95% confidence intervals (CIs) (15). This method provides a robust framework for identifying and quantifying shifts in disease dynamic.

Joinpoint regression analyses were performed by joinpoint regression program (Version 5.1.0; Statistical Methodology and Applications Branch, Surveillance Research Program, National Cancer Institute, available at https://surveillance.cancer.gov/joinpoint/). We employed the confidence interval overlap method to compare country-specific AAPCs with the global average (16). Specifically, we assessed whether the 95% CIs of each country’s AAPC overlapped with the global AAPC CI. Countries with non-overlapping CIs were classified as either significantly higher or significantly lower than the global mean. Data collation and visualizations were conducted using R software (Version 4.3.3). A threshold of 0.05 for the two-tailed *p*-value was employed to establish statistical significance.

## Result

### Global trends in ASIR, ASPR, ASMR, and ASDR of idiopathic epilepsy

In 2021, there were 1,227,191.11 (95% UI: 786,363.03 to 1,734,488.13) incident idiopathic epilepsy (Table 1) and 6,095,769.80 (95% UI: 4,272,870.83 to 8,270,631.23) prevalent idiopathic epilepsy (Table 2), 3564496.91 (95% UI: 2,700,943.67 to 4,753,410.38) DALYs due to idiopathic epilepsy (Table 3), and 18,171.15 (95% UI: 13,891.14 to 21,418.35) deaths due to idiopathic epilepsy (Table 4). The ASIR increased by 0.27% (95% CI: 0.24 to 0.29) for incidence from 55.74 to 100,000 population in 1990 to 61.35 per 100,000 in 2021. The ASPR (AAPC = −0.03; 95% CI: −0 0.06 to 0.01), ASMR (AAPC = −1.60; 95% CI: −1.83 to −1.36), and ASDR (AAPC = −1.01; 95% CI: −1.10 to −0.92) are decreased. In terms of gender, ASIR, ASPR, ASMR and ASDR for idiopathic epilepsy were higher in boys than girls. And, the ASIR for both boys and girls shows an increasing trend from year to year (AAPC>0, *p*<0.05). However, it is encouraging to note that the boys’ ASPR, ASMR, and ASDR, are all showing a downward trend from 1990–2021 (AAPC<0, *p*<0.05). Such a situation was then consistent with the results in girls’ ASMR and ASDR (AAPC<0, *p*<0.05).

**Table 1:** Incidence of idiopathic epilepsy in children (0–14 years) at the global, SDI, and regional levels.

Prevalence	1990		2021		1990–2021
	Cases no. (95% UI)	ASIR/100,000 (95% CI)	Cases no. (95% UI)	ASIR/100,000 (95% CI)	AAPC (%) (95% CI)
Global	971368.10 (624226.22 to 1368635.97)	55.74 (33.54 to 85.03)	1227191.11 (786363.03 to 1734488.13)	61.35 (37.76 to 94.00)	0.27 (0.24 to 0.29)*
Sex					
Boy	526423.08 (339990.29 to 738098.38)	58.76 (35.46 to 89.36)	664338.11 (430058.92 to 927297.35)	64.41 (40.11 to 98.24)	0.24 (0.22 to 0.26)*
Girl	444945.02 (282785.35 to 631061.90)	52.55 (31.23 to 80.66)	562852.99 (356915.46 to 807527.81)	58.09 (35.18 to 89.49)	0.29 (0.25 to 0.33)*
Socio-demographic index					
Low SDI	150783.90 (80733.23 to 230378.87)	47.24 (24.08 to 76.34)	306197.53 (185486.33 to 438447.02)	47.22 (27.32 to 72.54)	0.03 (−0.02 to 0.07)
Low-middle SDI	255940.09 (151098.03 to 382225.39)	39.25 (21.78 to 60.59)	341687.50 (215945.36 to 481879.10)	42.37 (25.87 to 62.96)	0.25 (0.16 to 0.34)*
Middle SDI	312668.14 (193388.77 to 442722.43)	38.99 (23.19 to 58.20)	334091.53 (209540.17 to 485226.46)	42.58 (25.73 to 63.65)	0.24 (0.21 to 0.28)*
High-middle SDI	131788.23 (82694.18 to 188452.97)	34.90 (21.00 to 52.30)	122321.80 (72912.35 to 188315.93)	39.07 (22.09 to 61.00)	0.29 (0.25 to 0.33)*
High SDI	119259.76 (76300.87 to 178953.18)	46.81 (27.23 to 71.51)	121917.97 (67742.29 to 188732.86)	51.81 (27.94 to 80.37)	0.30 (0.27 to 0.33)*
Regions					
African Region	183555.72 (106429.15 to 272669.09)	78.27 (43.34 to 123.19)	377416.77 (233082.26 to 536154.64)	78.70 (46.31 to 120.81)	−0.01 (−0.09 to 0.07)
Eastern Mediterranean Region	100207.87 (54760.70 to 149274.78)	61.20 (32.14 to 97.98)	158481.41 (94009.38 to 234714.57)	64.14 (36.17 to 101.60)	0.11 (0.05 to 0.17) *
European Region	133519.31 (84422.12 to 192113.01)	68.47 (41.13 to 103.82)	120621.18 (72349.12 to 180581.70)	73.54 (41.20 to 116.64)	0.18 (0.11 to 0.25)*
Region of the Americas	169600.79 (106577.64 to 242016.41)	77.30 (46.30 to 118.24)	165834.23 (107000.99 to 241221.22)	74.73 (43.61 to 116.81)	−0.12 (−0.16 to −0.09) *
South-East Asia Region	224786.39 (129770.79 to 335974.28)	45.27 (24.80 to 71.45)	241268.39 (151735.44 to 347050.06)	46.53 (27.49 to 72.03)	−0.04 (−0.15 to 0.07)
Western Pacific Region	155574.91 (96537.08 to 223616.15)	36.54 (21.18 to 56.85)	160814.24 (99221.18 to 240495.77)	44.32 (25.64 to 69.61)	0.57 (0.48 to 0.66)*

^*^Significantly different from 0 at alpha = 0.05 (*p* < 0.05). ASIR, age-standardized incidence rate; SDI, Socio-demographic index; UI, Uncertainty interval; AAPC, average annual percentage change; CI, confidence interval.

**Table 2:** Prevalence of idiopathic epilepsy in children (0–14 years) at the global, SDI, and regional levels.

Prevalence	1990		2021		1990–2021
	Cases no. (95% UI)	ASPR/100,000 (95% CI)	Cases no. (95% UI)	ASPR/100,000 (95% CI)	AAPC (%) (95% CI)
Global	5284295.40 (3817984.14 to 7189787.91)	304.05 (205.32 to 428.35)	6095769.80 (4272870.83 to 8270631.23)	301.38 (203.91 to 426.31)	−0.03 (−0.06 to 0.01)
Sex					
Boy	2848076.83 (2070361.84 to 3858260.14)	318.98 (216.15 to 449.50)	3255634.05 (2280504.87 to 4392963.81)	311.93 (212.48 to 438.50)	−0.07 (−0.11 to −0.03)*
Girl	2436218.57 (1746527.12 to 3319648.48)	288.27 (194.77 to 407.22)	2840135.75 (1990562.57 to 3873303.65)	290.13 (193.84 to 413.09)	0.02 (−0.01 to 0.05)
Socio-demographic index					
Low SDI	748854.83 (427076.38 to 1143084.39)	217.36 (118.17 to 344.01)	1428966.96 (946166.79 to 2031393.92)	199.78 (129.52 to 292.83)	−0.18 (−0.22 to −0.15)*
Low-middle SDI	1434144.71 (893591.92 to 2027430.70)	205.53 (123.87 to 304.32)	1767888.11 (1223828.81 to 2428416.96)	199.84 (136.50 to 276.32)	−0.01 (−0.07 to 0.06)
Middle SDI	1766843.96 (1269227.44 to 2402943.17)	207.21 (143.55 to 293.78)	1693513.86 (1137384.05 to 2373846.36)	193.12 (131.49 to 276.42)	−0.10 (−0.15 to −0.06)*
High-middle SDI	756603.18 (522011.95 to 1015357.44)	187.68 (127.96 to 259.98)	622034.06 (395354.33 to 902791.66)	175.28 (112.67 to 256.87)	−0.10 (−0.13 to −0.07) *
High SDI	573080.54 (388721.81 to 796460.03)	201.87 (132.74 to 287.85)	578578.43 (367734.94 to 836862.50)	216.32 (133.15 to 323.89)	0.21 (0.17 to 0.25)*
Regions					
African Region	846434.03 (524909.14 to 1240598.78)	371.68 (221.58 to 563.59)	1714494.83 (1152388.08 to 2364354.21)	359.89 (233.02 to 519.67)	−0.08 (−0.12 to −0.04)*
Eastern Mediterranean Region	573612.32 (334249.04 to 820191.80)	352.98 (200.22 to 538.40)	817013.23 (541950.92 to 1181563.29)	329.69 (208.78 to 488.76)	−0.24 (−0.32 to −0.16)*
European Region	676219.69 (462931.12 to 926974.30)	343.56 (224.73 to 490.80)	579285.67 (375349.37 to 828138.59)	345.21 (212.59 to 513.62)	−0.01 (−0.10 to 0.08)
Region of the Americas	886419.97 (641772.67 to 1202849.39)	403.32 (272.07 to 574.18)	825260.87 (554663.13 to 1152699.69)	367.37 (237.52 to 542.39)	−0.30 (−0.41 to −0.18)*
South-East Asia Region	1332657.91 (844802.32 to 1883965.10)	268.86 (163.05 to 398.58)	1322773.60 (892988.86 to 1826276.32)	250.94 (165.03 to 357.03)	−0.28 (−0.42 to −0.15)*
Western Pacific Region	944728.51 (659983.60 to 1290174.19)	221.74 (147.81 to 318.90)	821582.40 (541995.20 to 1165301.85)	221.87 (142.54 to 320.71)	0.01 (−0.05 to 0.06)

*Significantly different from 0 at alpha = 0.05 (*p* < 0.05). ASPR, age-standardized prevalence rate; SDI, Socio-demographic index; UI, Uncertainty interval; AAPC, average annual percentage change; CI, confidence interval.

**Table 3:** DALYs of idiopathic epilepsy in children (0–14 years) at the global, SDI, and regional levels.

Mortality	1990		2021		1990–2021
	Cases no. (95% UI)	ASDR/100,000 (95% CI)	Cases no. (95% UI)	ASDR/100,000 (95% CI)	AAPC (%) (95% CI)
Global	4188139.99 (3112385.06 to 5405104.59)	240.12 (173.36 to 318.18)	3564496.91 (2700943.67 to 4753410.38)	178.16 (130.40 to 245.81)	−1.01 (−1.10 to −0.92)*
Sex					
Boy	2248460.81 (1656803.92 to 2864073.62)	250.93 (177.97 to 331.09)	1992084.99 (1502139.44 to 2646836.56)	193.10 (141.02 to 263.32)	−0.88 (−0.98 to −0.79)*
Girl	1939679.18 (1356503.91 to 2603661.73)	228.74 (151.47 to 319.00)	1572411.92 (1154334.26 to 2158681.08)	162.22 (115.08 to 228.22)	−1.14 (−1.24 to −1.05)*
Socio-demographic index					
Low SDI	766403.47 (540703.53 to 1065346.85)	235.18 (161.30 to 327.52)	1125372.09 (827950.97 to 1507060.74)	168.81 (119.76 to 226.32)	−1.02 (−1.13 to −0.91)*
Low-middle SDI	1305333.47 (921269.55 to 1726607.31)	197.92 (134.22 to 265.74)	1199708.93 (892916.64 to 1592917.60)	147.39 (105.42 to 198.15)	−0.93 (−1.04 to −0.81)*
Middle SDI	1345361.17 (994554.04 to 1727491.25)	172.66 (123.36 to 222.72)	802489.02 (577972.38 to 1159408.69)	96.93 (69.84 to 134.93)	−1.63 (−1.71 to −1.55)*
High-middle SDI	539690.96 (416585.31 to 689060.40)	147.43 (108.43 to 191.11)	251502.24 (171033.29 to 376966.45)	73.86 (50.22 to 109.46)	−1.96 (−2.07 to −1.86)*
High SDI	228474.72 (155098.91 to 344204.39)	84.32 (57.58 to 124.98)	182866.84 (110649.71 to 305005.65)	70.49 (43.05 to 115.10)	−0.52 (−0.60 to −0.43)*
Regions					
African Region	691231.34 (501721.93 to 936350.97)	294.01 (200.90 to 417.02)	1136758.55 (824043.21 to 1547266.45)	237.19 (167.23 to 332.01)	−0.75 (−0.83 to −0.67)*
Eastern Mediterranean Region	492017.36 (357284.18 to 662331.77)	299.35 (208.97 to 417.96)	571479.10 (431983.92 to 763184.42)	231.46 (168.62 to 316.13)	−0.89 (−0.98 to −0.80)*
European Region	356139.74 (261052.74 to 487418.44)	182.17 (130.12 to 254.73)	233446.54 (159807.61 to 348167.24)	140.40 (94.79 to 212.38)	−0.84 (−0.96 to −0.73)*
Region of the Americas	473660.94 (347400.43 to 647186.74)	215.96 (156.71 to 305.10)	351781.30 (249391.35 to 518776.39)	158.04 (109.14 to 236.10)	−0.99 (−1.15 to −0.84)*
South-East Asia Region	1268437.11 (832171.74 to 1675697.47)	255.76 (163.25 to 349.14)	899309.49 (644143.66 to 1216220.24)	174.57 (117.02 to 247.02)	−1.27 (−1.44 to −1.10)*
Western Pacific Region	895376.63 (673628.17 to 1128192.83)	210.47 (151.81 to 273.11)	365568.70 (258178.33 to 547909.02)	99.48 (68.71 to 148.93)	−2.45 (−2.58 to −2.33)*

*Significantly different from 0 at alpha = 0.05 (*p* < 0.05). DALYs, disability-adjusted life-years; ASDR, age-standardized DALYs rate; SDI, Socio-demographic index; UI, Uncertainty interval; AAPC, average annual percentage change; CI, confidence interval.

**Table 4:** Mortality of idiopathic epilepsy in children (0–14 years) at the global, SDI, and regional levels.

Mortality	1990		2021		1990–2021
	Cases no. (95% UI)	ASMR/100,000 (95% CI)	Cases no. (95% UI)	ASMR/100,000 (95% CI)	AAPC (%) (95% CI)
Global	25767.96 (17566.63 to 30914.01)	1.47 (0.97 to 1.78)	18171.15 (13891.14 to 21418.35)	0.92 (0.67 to 1.12)	−1.60 (−1.83 to −1.36)*
Sex					
Boy	13801.22 (9130.86 to 16343.63)	1.54 (0.99 to 1.87)	10740.14 (7730.84 to 12993.43)	1.05 (0.74 to 1.31)	−1.28 (−1.43 to −1.14)*
Girl	11966.74 (6649.17 to 16310.51)	1.41 (0.73 to 1.94)	7431.01 (5101.17 to 9185.22)	0.78 (0.52 to 1.00)	−1.99 (−2.17 to −1.82)*
Socio-demographic index					
Low SDI	5225.41 (3420.90 to 6758.57)	1.63 (1.02 to 2.19)	6712.40 (4908.58 to 8302.00)	1.04 (0.72 to 1.34)	−1.40 (−1.74 to −1.06)*
Low-middle SDI	8466.81 (5015.98 to 10600.20)	1.31 (0.73 to 1.72)	6740.11 (4811.53 to 8160.25)	0.87 (0.58 to 1.13)	−1.38 (−1.63 to −1.12)*
Middle SDI	7944.58 (5566.36 to 9202.51)	1.08 (0.70 to 1.31)	3236.62 (2571.17 to 3725.63)	0.42 (0.31 to 0.50)	−2.81 (−3.03 to −2.59)*
High-middle SDI	3298.58 (2563.58 to 3895.35)	0.95 (0.68 to 1.18)	957.03 (826.89 to 1136.47)	0.29 (0.24 to 0.36)	−3.39 (−3.82 to −2.97)*
High SDI	818.75 (762.80 to 901.94)	0.32 (0.29 to 0.36)	513.45 (472.83 to 548.34)	0.21 (0.18 to 0.23)	−1.32 (−1.52 to −1.12)*
Regions					
African Region	3967.35 (2918.37 to 5039.13)	1.64 (1.17 to 2.11)	5710.46 (4290.01 to 7007.80)	1.18 (0.86 to 1.47)	−1.13 (−1.29 to −0.98)*
Eastern Mediterranean Region	3154.18 (2174.56 to 3978.44)	1.91 (1.26 to 2.50)	3463.40 (2739.78 to 4279.35)	1.41 (1.07 to 1.80)	−1.04 (−1.30 to −0.77)*
European Region	1725.26 (1443.63 to 2003.14)	0.89 (0.72 to 1.06)	973.04 (809.26 to 1119.63)	0.59 (0.48 to 0.70)	−1.30 (−1.45 to −1.14)*
Region of the Americas	1926.09 (1796.95 to 2065.85)	0.88 (0.81 to 0.95)	1288.52 (1070.86 to 1549.43)	0.60 (0.48 to 0.72)	−1.37 (−1.79 to −0.94)*
South-East Asia Region	8531.54 (4442.94 to 11149.52)	1.72 (0.88 to 2.34)	5233.57 (3323.51 to 6948.65)	1.03 (0.62 to 1.41)	−1.67 (−2.08 to −1.26)*
Western Pacific Region	6421.56 (4421.80 to 7691.04)	1.51 (1.02 to 1.87)	1480.94 (1226.59 to 1929.33)	0.41 (0.33 to 0.53)	−4.14 (−4.57 to −3.70)*

*Significantly different from 0 at alpha = 0.05 (*p* < 0.05). ASMR, age-standardized mortality rate; SDI, Socio-demographic index; UI, Uncertainty interval; AAPC, average annual percentage change; CI, confidence interval.

### Regional trend in ASIR, ASPR, ASMR, and ASDR of idiopathic epilepsy in five SDI regions

The number of incident idiopathic epilepsy, prevalent idiopathic epilepsy, idiopathic epilepsy-related deaths and idiopathic epilepsy -related DALYs in 2021 all were highest in Low-middle SDI region (Tables 1–4). In 2021, the highest ASIR and ASPR occurred in High SDI region. Moreover, Low SDI region had the highest ASMR and ASDR (Tables 1–4). The greatest increase of ASIR (AAPC = 0.30; 95% CI: 0.27 to 0.33) and ASPR (AAPC = 0.21; 95% CI: 0.17 to 0.25) both were in High SDI regions (Tables 1, 2). The ASMR and ASDR decreased across five SDI region; the greatest decline of ASMR (AAPC = −3.39; 95% CI: −3.82, −2.97) and ASDR (AAPC = −1.96; 95% CI: −2.07 to −1.86) were all in High-middle SDI region (Tables 3, 4).

### Regional trend in ASIR, ASPR, ASMR, and ASDR of idiopathic epilepsy in 6 regions

At the regional level (Tables 1–4), the African Region exhibited the highest ASIR (78.70; 95% CI: 46.31 to 120.81) and ASDR (237.19; 95% CI: 167.23 to 332.01); and the lowest ASIR (5.21; 95% CI: 3.19 to 7.60) were noticed in Western Pacific Region; the Region of the Americas has the highest ASPR (367.37; 95% CI: 237.52 to 542.39), the lowest ASPR (221.87; 95% CI: 142.54 to 320.71) were showed in Western Pacific Region; the Eastern Mediterranean Region has the highest ASMR (1.41; 95% CI: 1.07 to 1.80), and the European Region shows the lowest ASMR (0.59; 95% CI: 0.48 to 0.70). In addition, the Western Pacific Region had the largest increase in ASIR (AAPC = 0.57; 95% CI: 0.48 to 0.66) for idiopathic epilepsy in children; the Region of the Americas has the most significant decrease in ASPR (AAPC = −0.30; 95% CI: −0.41 to −0.18). Meanwhile, Western Pacific Region experienced the most significant decrease in ASMR (AAPC = −4.14; 95% CI: −4.57 to −3.70) and ASDR (AAPC = −2.45; 95% CI: −2.58 to −2.33).

### National trend in ASIR, ASPR, ASMR, and ASDR of idiopathic epilepsy

**Figure 1:**
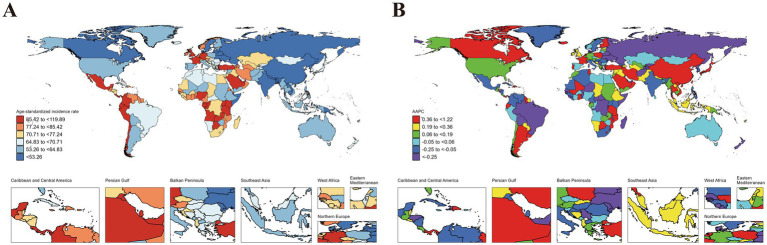
ASIR of idiopathic epilepsy in children (0–14 years) at the national level in 2021 **(A)**, and its changing trends from 1990 to 2021 **(B)**.

**Figure 2:**
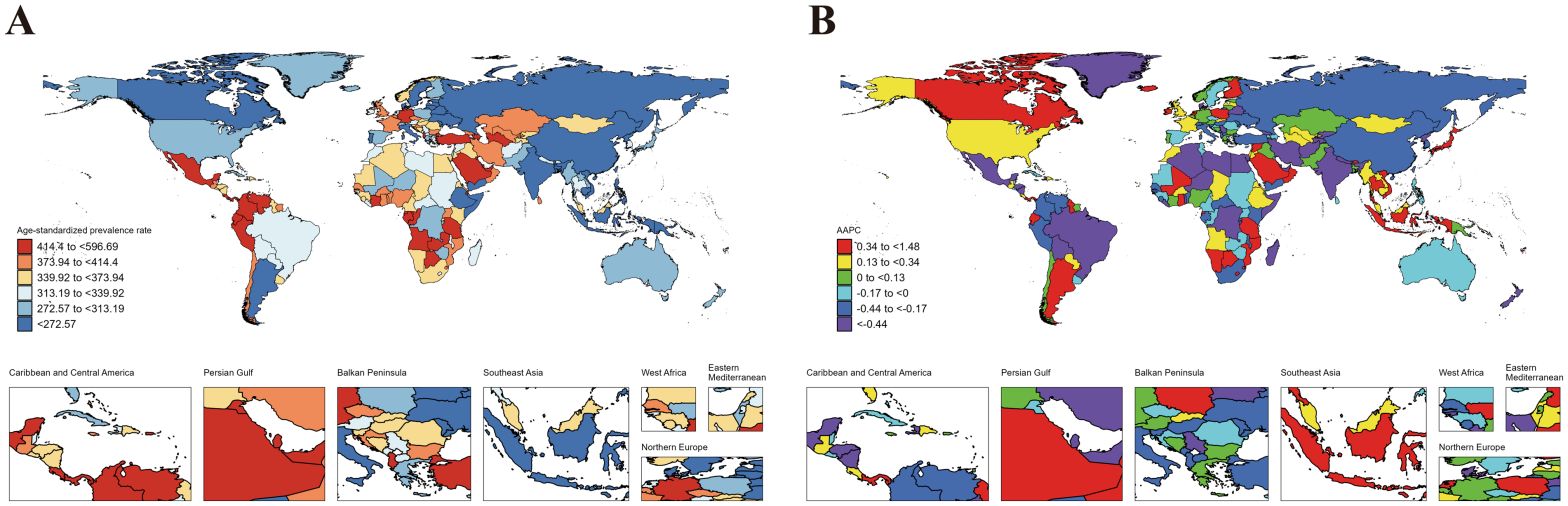
ASPR of idiopathic epilepsy in children (0–14 years) at the national level in 2021 **(A)**, and its changing trends from 1990 to 2021 **(B)**.

**Figure 3:**
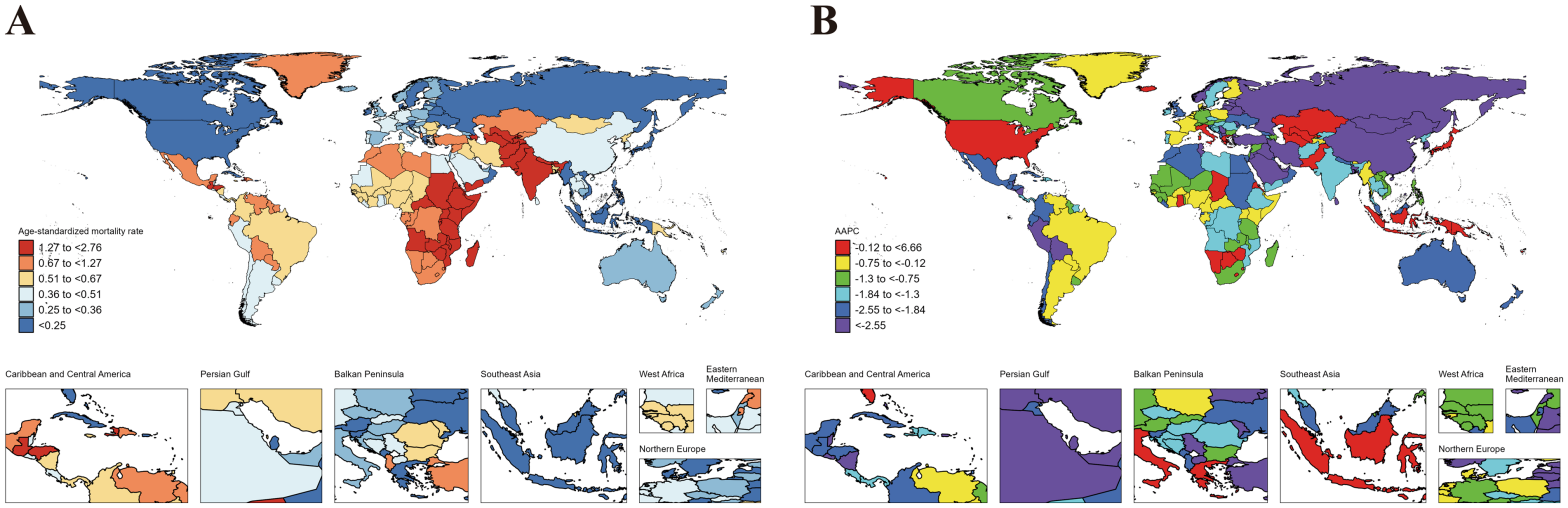
ASMR of idiopathic epilepsy in children (0–14 years) at the national level in 2021 **(A)**, and its changing trends from 1990 to 2021 **(B)**.

**Figure 4:**
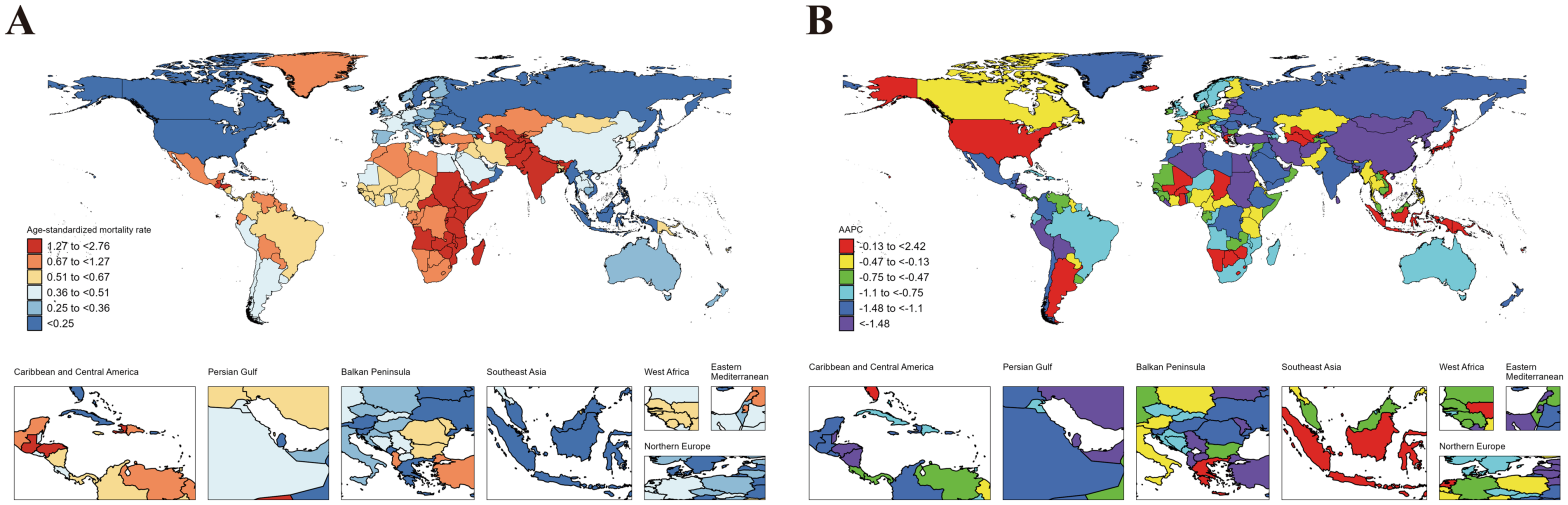
ASDR of idiopathic epilepsy in children (0–14 years) at the national level in 2021 **(A)**, and its changing trends from 1990 to 2021 **(B)**.

Variations in ASIR, ASPR, ASMR and ASDR were observed across 204 countries and territories (Figures 1A–4A). Among them, Antigua and Barbuda (99.25; 95% CI: 26.88 to 185.32), Mexico (98.28; 95% CI: 56.44 to 155.26), and Angola (98.28; 95% CI: 24.28 to 188.43) showed the highest ASIR, while the lowest ASIR were in Dominica (104.18; 95% CI: 30.98 to 191.52), Zambia (105.41; 95% CI: 23.71 to 212.57), and Gabon (111.26; 95% CI: 26.37 to 215.83). For ASPR, Equatorial Guinea (596.69; 95% CI: 148.49 to 1071.43) retained the top position, followed by Gabon (546.44; 95% CI: 122.91 to 985.31), and Ecuador (545.26; 95% CI: 170.21 to 945.21); while the lowest ASPR were observed in Democratic People’s Republic of Korea (173.52; 95% CI: 45.50 to 317.65), Solomon Islands (196.95; 95% CI: 45.72 to 384.15), and China (198.89; 95% CI: 124.93 to 291.55). In terms of ASMR, Tajikistan (2.76; 95% CI: 1.71 to 4.28) emerged as the country/region with the highest rate, followed by South Sudan (2.69; 95% CI: 1.76 to 4.00), and United Republic of Tanzania (2.53; 95% CI: 1.65 to 3.73); while the lowest ASMR were in Viet Nam (0.03; 95% CI: 0.00 to 0.12), San Marino (0.05; 95% CI: 0.03 to 0.07), and Northern Mariana Islands (0.09; 95% CI: 0.06 to 0.13). Moreover, Taiwan (Province of China) (99.28; 95% CI: 41.48 to 198.72), Northern Mariana Islands (98.48; 95% CI: 28.19 to 209.32), and China (96.39; 95% CI: 66.97 to 141.64) showed the highest ASDR, while the lowest ASDR were shown in New Zealand (100.54; 95% CI: 49.52 to 186.98), Slovenia (100.73; 95% CI: 34.06 to 224.87), and Guam (102.44; 95% CI: 31.52 to 213.02) (Figures 1A–4A).

The largest increase in ASIR was observed in Netherlands (1.22; 95% CI: 1.06 to 1.39), followed by Equatorial Guinea (1.11; 95% CI: 1.01 to 1.20), and Botswana (0.86; 95% CI: 0.80 to 0.92), while the largest decreases occurred in Togo (−0.94; 95% CI: −1.10 to −0.79), Liberia (−0.84; 95% CI: −1.08 to −0.60), and Tajikistan (−0.77; 95% CI: −0.91 to −0.62). The largest increase in ASPR was in Equatorial Guinea (1.48; 95% CI: 1.35 to 1.61), followed by Botswana (1.21; 95% CI: 1.12 to 1.31), and Netherlands (1.04; 95% CI: 0.81 to 1.26); while the largest decreases were noticed in Democratic People’s Republic of Korea (−1.35; 95% CI: −1.43 to −1.28), Liberia (−1.20; 95% CI: −1.31 to −1.09), and Togo (−1.17; 95% CI: −1.32 to −1.01). The countries with the largest increase in the ASMR were Tokelau (6.66; 5.43 to 7.9195% CI:), Niue (4.16; 95% CI: 2.47 to 5.87), and Northern Mariana Islands (2.45; 95% CI: 1.27 to 3.64); while the largest decreases were noticed in Latvia (−5.85; 95% CI: −6.98 to −4.70), Estonia (−4.73; 95% CI: −5.48 to −3.98), and China (−4.35; 95% CI: −4.77 to −3.94). The countries with the largest increase in the ASDR were Tokelau (2.42; 95% CI: 1.27 to 3.58), Niue (1.52; 95% CI: 0.91 to 2.13), and Lesotho (0.73; 95% CI: 0.52 to 0.95); while the largest decreases were noticed in China (−3.00; 95% CI: −3.18 to −2.82), Iran (Islamic Republic of) (−2.51; 95% CI: −2.69 to −2.32), and Estonia (−2.42; 95% CI: −2.67 to −2.16) (Figures 1B–4B).

### Comparison of country-specific AAPCs against global averages

The analysis revealed significant disparities in the AAPCs for ASIR, ASPPR, ASMR, and ASDR across countries when compared to global averages. For ASIR, several countries exhibited markedly higher AAPCs than the global average (0.27, 95% CI: 0.24 to 0.29), with the top performers being Netherlands (1.22, 95% CI: 1.06 to 1.39), Equatorial Guinea (1.11, 95% CI: 1.01 to 1.20), and Botswana (0.86, 95% CI: 0.80 to 0.92). Conversely, the lowest ASIR values were observed in Togo (−0.94, 95% CI: −1.10 to −0.79), Liberia (−0.84, 95% CI: −1.08 to −0.60), and Tajikistan (−0.77, 95% CI: −0.91 to −0.62). For ASPR, the global AAPC was −0.03 (95% CI: −0.06 to 0.01). Countries with the highest ASPR increases included Equatorial Guinea (1.48, 95% CI: 1.35 to 1.61), Botswana (1.21, 95% CI: 1.12 to 1.31), and Netherlands (1.04, 95% CI: 0.81 to 1.26). In contrast, Democratic People’s Republic of Korea (−1.35, 95% CI: −1.43 to −1.28), Liberia (−1.20, 95% CI: −1.31 to −1.09), and Togo (−1.17, 95% CI: −1.32 to −1.01) showed the lowest ASPR trends, which may reflect underdiagnosis or limited disease management. The global AAPC for ASMR was −1.60 (95% CI: −1.83 to −1.36), reflecting an overall decline. The steepest reductions were observed in Latvia (−5.85, 95% CI: −6.98 to −4.70), Estonia (−1.05, 95% CI: −1.20 to −0.90), and China (−4.35, 95% CI: −4.77 to −3.94). However, some countries experienced increases in ASMR, including Tokelau (6.66, 95% CI: 5.43 to 7.91), Niue (4.16, 95% CI: 2.47 to 5.87), and Northern Mariana Islands (2.45, 95% CI: 1.27 to 3.64), indicating challenges in addressing critical health issues. For ASDR, the global AAPC was −1.01 (95% CI: −1.10 to −0.92). Countries with the highest increases included Tokelau (2.42, 95% CI: 1.27 to 3.58), Niue (1.52, 95% CI: 0.91 to 2.13), and Lesotho (0.73, 95% CI: 0.52 to 0.95). In contrast, the steepest declines were observed in China (−3.00, 95% CI: −3.18 to −2.82), Iran (Islamic Republic of) (−2.51, 95% CI: −2.69 to 2.32), and Estonia (−2.42, 95% CI: −2.67 to −2.16). (Details are provided in the Supplementary materials).

## Discussion

The global trends in the incidence, prevalence, mortality, and disability-adjusted life years of idiopathic epilepsy among children from 1990 to 2021 reveal significant patterns and regional variations, reflecting both advancements in healthcare and persistent challenges in managing this neurological disorder, which is consistence with the previous study (10, 17). The ASIR showed a slight increase trend. In contrast, the ASPR, ASMR, and ASDR all decreased over the same period. The observed increase in the ASIR is likely multifactorial. While improvements in diagnostic capabilities and greater awareness of epilepsy are plausible contributors (18), the Global Burden of Disease (GBD) estimates also incorporate modeling to account for undiagnosed cases, reducing the influence of underreporting. This suggests that other factors, such as increased environmental, genetic, or socioeconomic risk exposures, might also play a role. Additional studies that delve into these dimensions are necessary to validate this hypothesis. Conversely, the decrease in the ASPR, ASMR, and ASDR suggests that there have been significant improvements in the management and treatment of idiopathic epilepsy (19, 20). These improvements may be attributed to better access to antiepileptic medications, increased availability of specialized care, and enhanced support systems for children and families affected by the disorder (21). This decline in mortality and overall disease burden is promising and indicates that ongoing public health initiatives and healthcare advancements are positively impacting outcomes for children with epilepsy. Consistent with previous studies (22), we found that boys have higher age-standardized incidence (ASIR), prevalence (ASPR), mortality (ASMR), and DALYs (ASDR) rates for idiopathic epilepsy compared to girls. This gender difference may be influenced by underlying biological, genetic, and possibly environmental factors that increase susceptibility to epilepsy in boys. However, the exact mechanisms remain to be fully elucidated and warrant further investigation.

Significant regional disparities emerge when examining trends across the five SDI regions. The low-middle SDI region continues to bear the heaviest burden of incident and prevalent idiopathic epilepsy cases, as well as the highest number of deaths and DALYs in 2021. This persistent burden underscores the healthcare challenges faced by lower-middle-income regions, including limited access to quality healthcare, diagnostic tools, and antiepileptic treatments (23). In contrast, high SDI regions show the highest ASIR and ASPR, reflecting improved diagnostic capabilities and more comprehensive reporting systems (24), the Netherlands, a prime example, boasts superior diagnostic and reporting capabilities, alongside substantial healthcare investment. Between 2019 and 2021, health spending in the Netherlands increased by over 12% in real terms (25). Characterized by universal health care coverage and a foundation of regulated competition, the Netherlands health system is recognized by the (37) as one of the world’s leading healthcare systems in terms of accessibility, equity, and clinical outcomes (26). However, this also reveals that rising incidence and prevalence rates in high SDI regions may be attributed to improved diagnostic accuracy, better public awareness, and greater reporting of previously underreported cases (24), it is important to recognize that GBD estimates aim to adjust for undiagnosed cases using robust statistical methods. While high SDI regions have experienced increases in ASIR and ASPR, the most significant declines in ASMR and ASDR are seen in high-middle SDI regions. This suggests that these regions have made substantial progress in reducing epilepsy-related mortality through better healthcare infrastructure, enhanced access to treatment, and more effective public health interventions. These improvements emphasize the importance of strengthening healthcare systems and addressing disease burden through targeted prevention and management strategies.

The African Region reports the highest ASIR and ASDR, indicating a severe burden of idiopathic epilepsy. These high rates might be partially explained by underestimation in other regions where case identification and reporting may be less comprehensive, despite GBD modeling efforts to correct for this. In these areas, challenges in healthcare infrastructure, limited access to diagnostic tools, antiepileptic drugs, and specialized therapies such as physical, occupational, and speech therapy, and possibly higher rates of comorbid conditions such as infections and malnutrition (27). The need for improved healthcare infrastructure, diagnostic tools, and access to treatments is critical for alleviating the burden of the disease (28). Strengthening public health systems in this region will be vital to improving outcomes for children with epilepsy (29).

The Western Pacific Region stands out with its relatively low ASIR and the most significant declines in both mortality and death rates. This region serves as an example of successful public health interventions, healthcare improvements, and early diagnosis systems. The strategies implemented in countries such as Japan and Australia, which include better healthcare access, effective treatment protocols, and early intervention, could serve as models for other regions facing higher disease burdens, demonstrating the effectiveness of improving healthcare access and public health initiatives. For example, the Australian healthcare system offers universal coverage to all residents, striving to ensure equitable, accessible, safe, and high-quality healthcare for its population (30). Meanwhile, Japan’s *Health Care 2035 Vision* aims to create a sustainable, responsive, and equitable healthcare system that improves health outcomes and contributes to both national and global prosperity (31).

In the Region of the Americas, the prevalence of idiopathic epilepsy remains high, but significant improvements in management and treatment have been made (32). Continued investments in healthcare infrastructure, public health education, and treatment accessibility will be essential in further reducing the burden of epilepsy. Enhanced public awareness campaigns and better healthcare delivery models will be crucial in improving long-term outcomes for children with epilepsy in this region (33). The Eastern Mediterranean Region faces higher ASMR and ASDR, indicating a pressing need for enhanced access to specialized care and better management strategies to reduce mortality and improve the quality of life for children with epilepsy (34, 35). This region is often hindered by political instability and fragmented healthcare systems, making access to effective treatments challenging. Addressing these issues will be crucial to improving outcomes for children affected by epilepsy.

At the national level, trends in ASIR, ASPR, ASMR, and ASDR underscore vast disparities in epilepsy burden across 204 countries and territories. Countries with the highest rates of epilepsy-related incidence and mortality often face significant challenges, including limited healthcare resources, inadequate public awareness, and insufficient medical infrastructure. These countries need to focus on strengthening their healthcare systems, improving awareness, and expanding access to medical care and antiepileptic drugs (36). Conversely, countries with lower rates of epilepsy-related incidence and mortality typically have more robust healthcare systems, earlier diagnosis, and better access to treatment. These nations, such as those in Western Europe and North America, serve as models for how comprehensive healthcare infrastructure, public health initiatives, and education can lead to better outcomes for children with epilepsy. However, countries such as the Netherlands and Equatorial Guinea, which have seen increases in incidence and prevalence, should focus on strengthening healthcare systems to ensure that the growing number of epilepsy cases is effectively managed. Adequate resources must be allocated for treatment, prevention, and research in these regions to manage the rising burden. Furthermore, enhancing collaboration between clinical and community programs, as well as between public health practitioners, epilepsy care providers, and social service agencies, can help bridge gaps in healthcare access for children (28).

## Conclusion

Trends in idiopathic epilepsy among children show both progress and ongoing challenges. Some regions have reduced mortality and disability, while others still face high incidence and prevalence rates. Disparities between high and low SDI regions underscore the need for targeted interventions, improved healthcare access, and better resource allocation. Strengthening healthcare systems, enhancing diagnostic and treatment capabilities, and raising awareness, especially in low and low-middle SDI regions, are crucial. Future research should focus on the causes of these disparities and develop strategies to ensure equitable healthcare for all affected children. Leveraging Global Burden of Disease data can help policymakers and healthcare providers better understand and address the condition’s impact globally.

### Limitation

A limitation of this study is the potential influence of regional disparities in diagnostic and reporting capabilities. While the GBD methodology adjusts for undiagnosed cases and reporting variability, residual biases may persist, particularly in regions with limited healthcare access or inconsistent data quality, potentially leading to underestimation of the true burden.

## Data Availability

The original contributions presented in the study are included in the article/Supplementary material, further inquiries can be directed to the corresponding authors.

## Footnotes

^1^ http://ghdx.healthdata.org/gbd-results-tool
